# Comparative Analysis of Labile and Stable Coagulation Factors in Fresh Frozen Plasma and Plasma Frozen 24 Hours After Phlebotomy: A Prospective Study

**DOI:** 10.7759/cureus.99158

**Published:** 2025-12-13

**Authors:** Nirupama Sahoo, Binay B Sahoo, Smita Mahapatra, Jagannath Sahoo, Susmita Behera

**Affiliations:** 1 Immuno-Hematology and Blood Transfusion, Kalinga Institute of Medical Sciences, Bhubaneswar, IND; 2 Transfusion Medicine, Srirama Chandra Bhanja Medical College and Hospital, Cuttack, IND; 3 Pathology, Srirama Chandra Bhanja Medical College and Hospital, Cuttack, IND

**Keywords:** coagulation factors, fresh frozen plasma (ffp), frozen plasma, international normalized ratio (inr), quality indicators of plasma

## Abstract

Background

Plasma contains coagulation factors, for which it is widely used for patients with coagulation deficiencies. The coagulation factors Factor V and Factor VIII are considered labile factors. The preparation methods and storage periods affect plasma quality. Fresh frozen plasma (FFP) is plasma prepared and frozen within six to eight hours after collection, and frozen plasma (FP24) is prepared and frozen from donor blood units after keeping them at 2-6°C for 16-24 hours after blood collection. The aim of this study is to measure both labile and stable coagulation factor levels and compare them between FFP and frozen plasma (FP24).

Materials and methods

A total of 100 plasma units were included. The FFPs were prepared as per our institutional standard operating procedure (SOP), and frozen plasma (F24) was prepared according to the Association for the Advancement of Blood and Biotherapies (AABB) guidelines. The coagulation parameters, such as prothrombin time (PT), international normalized ratio (INR), activated partial thromboplastin time (aPTT), and coagulation factors (V, VII, VIII, and fibrinogen), were estimated using a semi-automated coagulometer (Erba ECL-412) in our institution.

Result

The coagulation parameters, such as PT, INR, and aPTT, were increased, and the coagulation factors were decreased in FP24 plasma as compared to FFP on the day of preparation and after three and six months of storage. Statistically, some coagulation factors showed significant results, whereas others were insignificant.

Conclusion

The frozen plasma (FP24) can be used therapeutically in stable coagulation factor deficiencies, mostly fibrinogen deficiency, with proper guidelines.

## Introduction

Plasma, being the cell-free part of blood, is mostly composed of water, electrolytes, and proteins such as albumins, immunoglobulins, and coagulation factors. Plasma contains both labile coagulation factors, such as Factor V (F-V) and Factor VIII (F-VIII), and stable coagulation factors, such as Factor VII (F-VII) and fibrinogen. Fresh frozen plasma (FFP), frozen plasma, single-donor plasma, cryoprecipitate-reduced plasma, liquid plasma, pathogen-inactivated plasma, thawed plasma, and recovered plasma are different types of plasma prepared for clinical use.

Plasma that is separated and frozen at -18°C or colder within six to eight hours of whole blood collection is known as FFP, and if plasma is frozen between 8 and 24 hours after collection, it is known as plasma frozen 24 hours after phlebotomy, or frozen plasma (FP24) [1]. FFP is mostly indicated for replacement of single coagulation factor deficiencies for which recombinant factor concentrates are not available, or for multiple coagulation factor deficiency conditions such as acute disseminated intravascular coagulation (DIC), liver disease, and massive transfusion [2,3], as well as for therapeutic plasma exchange in thrombotic thrombocytopenic purpura (TTP). In some instances, it is used for warfarin reversal and in cardiopulmonary bypass surgery [4].

The F-VIII level significantly decreases in extracted plasma when blood is stored at 4°C for a short interval of time, whereas the activity levels of other coagulation factors remain unchanged [5]. The quality control criteria set forth by the Directorate General of Health Services (DGHS), Ministry of Health and Family Welfare, Government of India, require fibrinogen levels of 200-400 mg/bag and F-VIII activity of 0.7 units/mL [6].

The aim of this study was to measure and compare the levels of labile and stable coagulation factors between FFP and plasma prepared 24 hours after phlebotomy.

## Materials and methods

This was a prospective study conducted over a period of two years, from 2018 to 2020 (approval no. 445), in the Department of Transfusion Medicine, Srirama Chandra Bhanja (SCB) Medical College and Hospital, Cuttack, India.

Sample size

A total of 100 plasma units, 50 samples each from FFP and plasma frozen within 24 hours of phlebotomy (FP24), were included in this study. A 350 mL whole blood sample was collected in Top & Top-type triple bags from voluntary and replacement donors fulfilling the donor eligibility criteria as per the Drug and Cosmetic Rules, 1940.

Preparation of FFP

The whole blood units were prepared within six hours of collection by centrifugation in a Blood Bank centrifuge (Cryofuge 16, Thermo Fisher Scientific, Waltham, MA, USA) using a soft spin (1515 rpm at 22°C for 12 minutes), followed by heavy spin centrifugation (3536 rpm at 22°C for 8 minutes). Plasma was separated from the parent bag manually using a plasma expressor (Labtop Instruments Pvt. Ltd, Vasai, India) and frozen below -30°C in the blood bank deep freezer within eight hours of collection. The preparation of FFP was performed as per the standard operating procedure (SOP) in our institution.

Preparation of FP24

The whole blood units were kept in the Blood Bank refrigerator (Terumo Penpol, Thiruvananthapuram, Kerala) at 2-6°C for 16-20 hours. The whole blood units were then centrifuged in a Blood Bank centrifuge (Cryofuge 16, Thermo Fisher Scientific) using a soft spin (1515 rpm at 22°C for 12 minutes). Plasma was separated from the parent bag manually using a plasma expressor (Labtop Instruments Pvt. Ltd) and frozen in a deep freezer below -30°C.

Inclusion criteria

The plasma units, prepared from blood units collected from voluntary blood donors, after fulfilling the National AIDS Control Organization (NACO) eligibility criteria.

Exclusion criteria

Transfusion-transmitted infection-positive units, units with breakage or leakage, and lipemic or discolored plasma units were excluded from our study.

Coagulation studies

A 5 mL sample was collected aseptically from each plasma unit for coagulation factor estimation. The coagulation parameters, including prothrombin time (PT), activated partial thromboplastin time (APTT), F-V, F-VII, F-VIII, and fibrinogen, were measured on day 0 for FFP, day 1 for FP24, and after three and six months of storage below -30°C. The frozen units were thawed in a plasma thaw bath at 37°C before sample collection. The tests were performed using a semi-automatic coagulometer (Erba ECL 412) as per the manufacturer’s instructions, using reagents (Siemens Diagnostics, Marburg, Germany).

**Figure 1:**
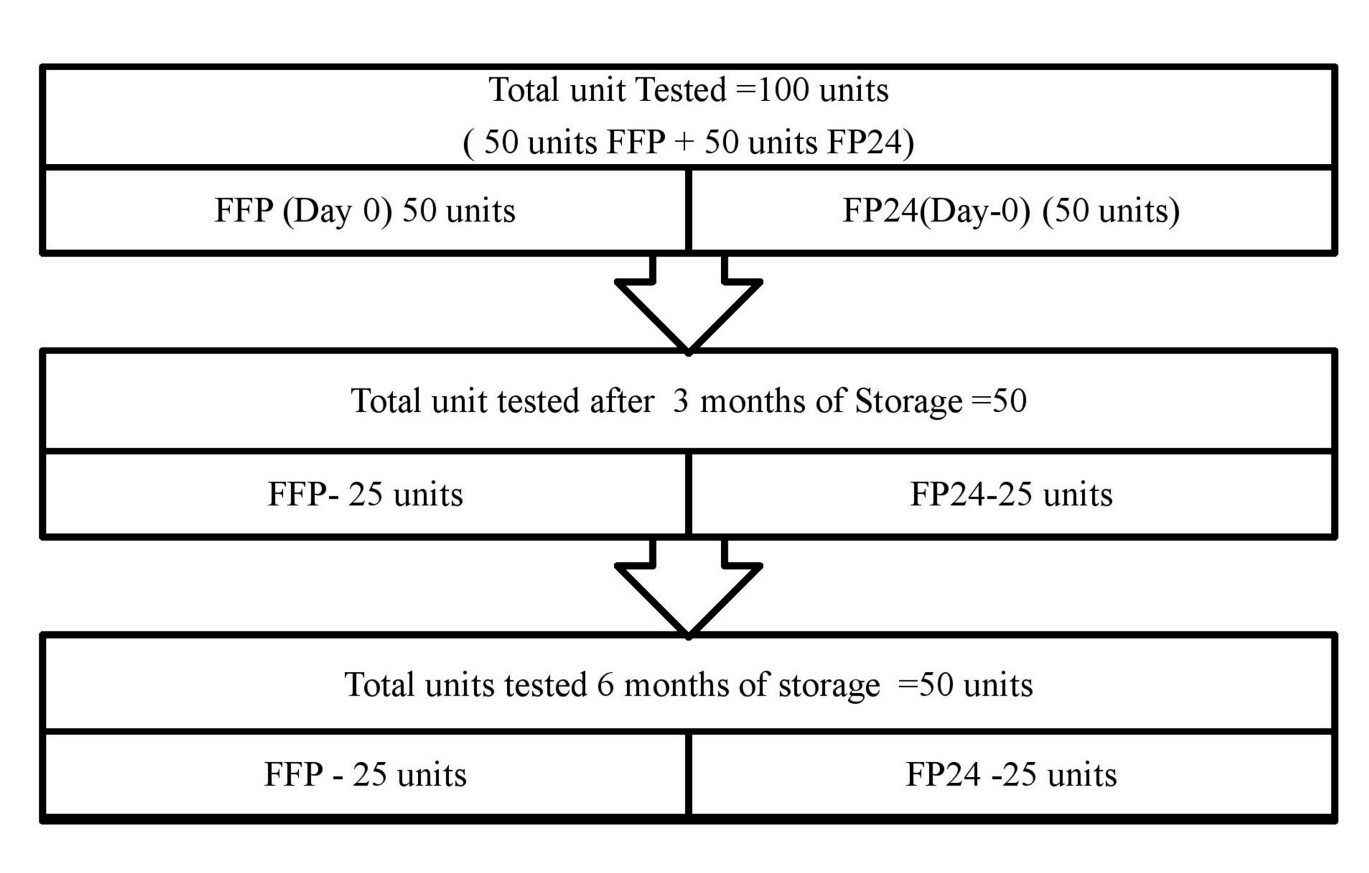
Distribution of subjects in coagulation factor testing FFP, Fresh frozen plasma

Statistical analysis

Data were collected in an Excel sheet (Microsoft® Corp., Redmond, WA, USA) and analyzed using IBM SPSS Statistics for Windows, Version 21 (Released 2012; IBM Corp., Armonk, NY, USA). We used descriptive statistics, such as percentage, mean, and standard deviation, and inferential statistics, such as the t-test, for statistical analysis. A p-value of <0.05 was considered significant.

## Results

This study included 100 plasma units prepared from both voluntary and replacement blood donors. Of these 100 units, 96 plasma units were collected from male donors and four units from female donors. The majority of plasma units were from 'O' blood group (46%) donors. The coagulation factor parameters on the day of preparation in FFP and FP24 are shown in Table 1.

**Table 1:** Comparison of coagulation factors between FFP and FP24 on the day of preparation The data are represented as mean ± SD (standard deviation). A p-value of <0.05 was considered significant. FFP, fresh frozen plasma; PT, prothrombin time; aPTT, activated partial thromboplastin time; INR, international normalized ratio

Coagulation factors	Day 0 (mean ± SD)	Day 1 (mean ± SD)	Change in percentage	p-value (t-statistic value)
PT (seconds)	12.16 ± 0.85	12.99 ± 0.62	7	0.001 (6.721)
INR	0.97 ± 0.08	1.04 ± 0.06	7	0.001 (4.950)
APTT (seconds)	30.77 ± 2.41	32.83 ± 2.86	6.6	0.002 (3.781)
F-V	93.49 ± 26.56	81.3 ± 19.91	13	0.01 (-2.590)
F-VII	94.85 ± 26.12	85.49 ± 21.93	10	0.055 (-1.866)
F-VIII	120.01 ± 33.07	84.1 ± 34.96	30	0.0001 (-5.290)
Fibrinogen (mg/dL)	260.18 ± 63.35	253.72 ± 54.38	2.5	0.585 (-0.593)

The mean coagulation parameters, including PT, international normalized ratio (INR), and APTT, were increased by 6.5%, 6%, and 8.2%, respectively, in FP24 compared to FFP plasma after three months of storage, as shown in Table 2.

**Table 2:** Comparison of coagulation parameters between FFP and FP24 plasma after three months of storage The data are represented as mean ± SD (standard deviation). A p-value of <0.05 was considered significant. FFP, fresh frozen plasma; PT, prothrombin time; aPTT, activated partial thromboplastin time; INR, international normalized ratio

Coagulation factors	FFP (mean ± SD)	FP24 (mean ± SD)	Change in percentage	p-value (t-statistic value)
PT (seconds)	13.74 ± 0.59	14.63 ± 0.82	6.5%	<0.001 (4.950)
INR	1.09 ± 0.03	1.16 ± 0.06	6%	1.00 (0.000)
APTT (seconds)	36.02 ± 2.88	38.99 ± 4.09	8.2%	0.004 (2.999)
F-V	81.43 ± 14.53	75.21 ± 17.25	7.6%	0.19 (-1.330)
F-VII	98.82 ± 24.9	83.48 ± 20.39	15.5%	0.017 (-2.486)
F-VIII	84.16 ± 13.32	64.47 ± 18.18	23.4%	<0.001 (-4.437)
Fibrinogen (mg/dL)	219.98 ± 21.42	215.24 ± 54.32	2.15%	0.67 (0.428)

The high levels of PT and INR in FP24 were statistically significant (p < 0.001 and p = 0.004), but APTT was not significant. On comparison of mean factor levels, F-V, F-VIII, and F-VII were 7.6%, 23.4%, and 15.5% lower, respectively, in FP24 compared to FFP plasma, whereas the fibrinogen level was only 2.5% lower. Both F-VIII and fibrinogen levels were statistically significant (p < 0.001 and p = 0.006).

After a storage period of six months, the mean PT, INR, and APTT in FP24 increased by 4.8%, 3.2%, and 3.3%, respectively, and the mean factor levels, such as F-V, F-VII, and F-VIII, decreased by 7.6%, 8.4%, and 24.9%, respectively, in comparison to FFP, whereas the fibrinogen level decreased slightly. The decrease in F-VIII was statistically significant (p = 0.001), as shown in Table 3.

**Table 3:** Comparison of coagulation parameters between FFP and FP24 plasma after six months of storage The data are represented as mean ± SD (standard deviation). A p-value of <0.05 was considered significant. FFP, fresh frozen plasma; PT, prothrombin time; aPTT, activated partial thromboplastin time; INR, international normalized ratio

Coagulation factors	FFP (mean ± SD)	FP24 (mean ± SD)	Change in percentage	p-value (t-statistic value)
PT (seconds)	15.63 ± 2.0	16.38 ± 1.3	4.8%	0.041 (2.096)
INR	1.25 ± 0.17	1.29 ± 0.1	3.2%	1.00 (0.000)
APTT (seconds)	39.08 ± 4.81	40.37 ± 4.49	3.3%	0.306 (1.034)
F-V	76.00 ± 16.49	70.20 ± 13.17	7.6%	0.162 (-1.422)
F-VII	70.84 ± 15.13	64.85 ± 9.52	8.4%	0.099 (-1.678)
F-VIII	61.95 ± 15.84	46.48 ± 15.48	24.9%	0.001 (-3.612)
Fibrinogen (mg/dL)	214.5 ± 31.74	213.82 ± 37.61	0.3%	0.919 (-0.102)

**Figure 2:**
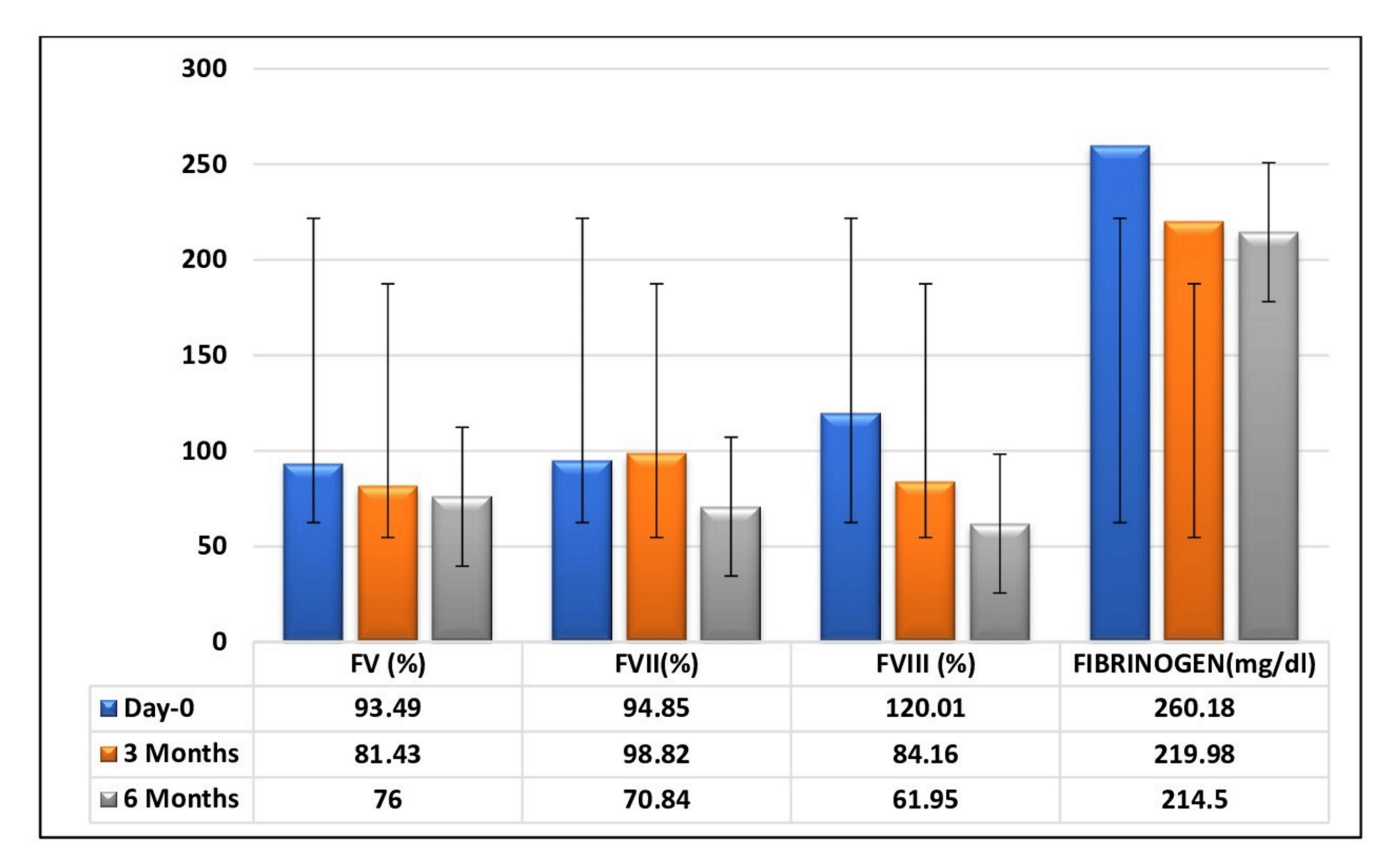
Change in mean coagulation factor levels of FFP in relation to the storage period The graph has been represented as mean values with error bars. FFP, fresh frozen plasma

**Figure 3:**
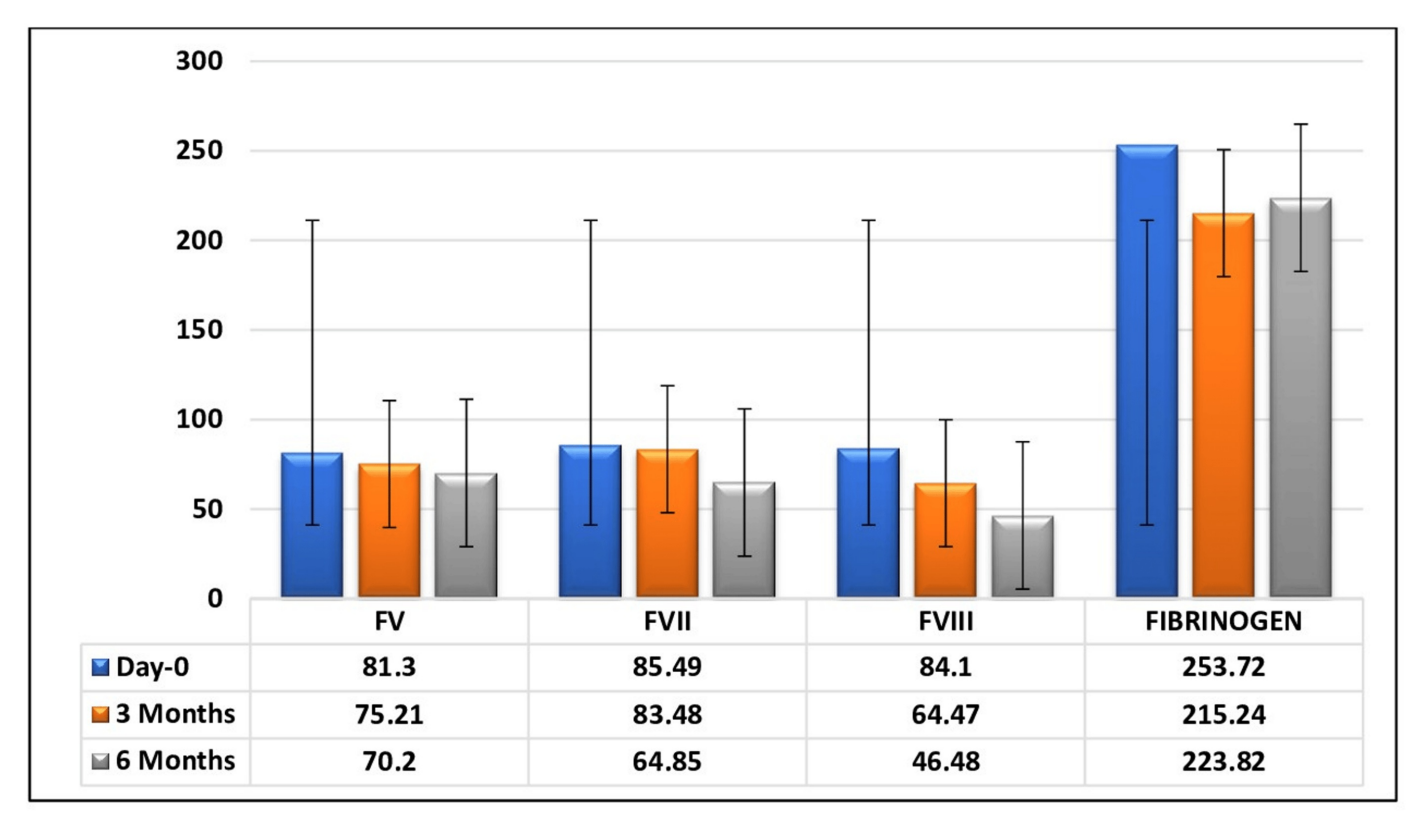
Change in mean coagulation factor level of FP24 in relation to the storage period The graph has been represented as mean values with error bars.

All the mean factor levels decreased during the progressive storage period, both in FFP (Figure 2) and FP24 plasma (Figure 3).

The mean coagulation factors in the O and non-O blood groups on the day of preparation are shown in Table 4.

**Table 4:** Change in mean coagulation factor levels between O and non-O blood groups in FFP and FP24 plasma The data are represented as mean ± SD (standard deviation). A p-value of <0.05 was considered significant. FFP, fresh frozen plasma

Coagulation factors	FFP			FP24		
	Group-O (mean ± SD)	Non-group-O (mean ± SD)	p-value (t-statistic value)	Group-O (mean ± SD)	Non-group-O (mean± SD)	p-value (t-statistic value)
F-V	87.87 ± 18.85	90.64 ± 29.85	0.72 (0.355)	80.15 ± 17.70	82.78 ± 22.75	0.67 (0.423)
F-VII	83.7 ± 20.42	101.12 ± 27.14	0.02 (2.368)	83.74 ± 21.35	87.74 ± 22.95	0.54 (0.606)
F-VIII	105.92 ± 34.81	127.94 ± 29.74	0.02 (2.363)	82.34 ± 31.26	86.29 ± 39.88	0.71 (0.362)
Fibrinogen (mg/dL)	257.08 ± 73.74	261.91 ± 57.90	0.79 (0.256)	252.93 ± 54.98	254.72 ± 54.87	0.91 (0.111)

All coagulation factor levels were higher in the non-O groups in both FFP and FP24 plasma, but this was statistically insignificant. The increases in coagulation factors in the non-O group in FP24 plasma were smaller compared to FFP.

## Discussion

The transfusion of plasma components is primarily for coagulation factor replacement. The change in coagulation factors depends on the preparation time and storage period. In our study, we compared the effect on coagulation between plasma prepared within six hours of collection (FFP) and plasma prepared by incubating collected blood at 2-6°C overnight, by measuring the coagulation parameters.

The mean PT (in seconds) in the FP24 group slightly increased by 7%, 6.5%, and 4.8% on day 0, after three months, and after six months of storage, respectively, compared to the FFP group. This increase was statistically significant on day 0 (p = 0.001) and after three months of storage (p < 0.001). This finding is in accordance with Li et al. [7] and Acker et al. [8], but contradicts Weisert and Jeremic [9], who reported a decrease in PT, and Sohmer et al. [10], who reported no change in mean PT. The mean INR values increased in FP24 on day 0 and throughout the storage period compared to FFP, but this was only significant on the day of preparation (p = 0.001). A similar finding was observed by Afifi et al. [11]. The mean APTT values also increased in FP24 in the present study compared to FFP, both on the day of preparation and throughout the storage period, which is in accordance with Afifi et al.'s study [11]. Similar results were noted by Feng et al. [12], but no change in APTT levels post-filtration was reported by Nilsson et al. [13].

The coagulation factors F-V, F-VII, F-VIII, and fibrinogen levels decreased in both FFP and FP24 during the storage period compared to the day of preparation. The F-V levels decreased by 13% in FP24 plasma on day 0 and by approximately 8% after three and six months of storage compared to FFP. This was similar to the findings of Cardigen et al. (15%) [5] and Youssef et al. (14%) [14], but higher than Dogra et al.'s study (6.5%) [15]. No change in F-V levels was observed by Naghadeh and Roudkenar [16], Alhumaidan et al. [17], Sheffield et al. [18], or Smith et al. [19]. The change in mean F-VII levels of 10%-15% in FP24 compared to FFP was statistically insignificant in our study. O’Neill et al. [20] reported an approximate 8% decrease in F-VII, and Scott et al. [21] observed a 15% decrease during storage of whole blood at 4°C for approximately 24 hours before plasma separation. Kakaiya et al. [22] reported that the change in F-VII levels was statistically significant.

F-VIII and fibrinogen levels are considered the most important quality indicators for plasma use. Good-quality plasma should have 0.7 units/mL of F-VIII and at least 200 mg per bag of fibrinogen. In our study, FP24 plasma maintained F-VIII levels only on the day of preparation, whereas FFP retained its value throughout the storage period. Fibrinogen levels were maintained throughout the storage period in both groups. Compared to FFP, F-VIII levels decreased by 30% in FP24 plasma. Our results were similar to O’Neill et al. (33%) [20] and Alakech et al. (29%) [23], but higher than Perkins et al. (16%) [24]. Although the changes in fibrinogen levels were statistically insignificant, F-VIII showed a significant decrease, which was also reported by Smith et al. [19] and Agus et al. [25]. The greater loss of F-VIII in FP24 may be due to increased activation of coagulation F-VIII with longer storage at 4°C after whole blood collection. Fibrinogen levels showed only a 2.5% decrease in FP24 plasma compared to FFP in our study. This result was similar to Alhumaidan et al. [17], who observed a 1.81% decrease, but larger losses of 12% and 29% were reported by Cardigan et al. [5] and Afifi et al. [11], respectively.

The relationship of coagulation in FFP and FP24 plasma between O and non-O blood groups was included in our study. Higher coagulation factor levels were observed in non-O blood group donors in both plasma groups compared to FFP. Statistically, the labile F-VIII showed a significant result (p = 0.02) in the FFP group. The higher F-VIII levels in non-O group donors were comparable to those reported by Wahlberg et al. [26], Favaloro et al. [27], and Subramaniyan et al. [28]. However, among non-O blood group plasma, FFP showed higher coagulation factor levels compared to FP24 on the day of preparation.

The study might have been more stringent if we had included a larger number of plasma units for coagulation factor estimation and a longer storage period. We could not establish the effect of coagulation parameters among male and female donors due to the inadequate number of female donations.

## Conclusions

Based on the findings, plasma prepared by incubating collected blood at 2-6°C overnight (FP24) showed greater loss of the labile coagulation factor F-VIII compared to other coagulation factors and to FFP. Although fibrinogen levels decreased only negligibly, their quality was maintained during the storage period. Therefore, we recommend that FP24 plasma may be used for the replacement of stable coagulation factors, particularly fibrinogen. In our country, FFP should be widely used, as there are no guidelines for FP24 plasma preparation and use. Proper guidelines are needed to establish FP24 plasma in setups where blood centers are located far from the site of blood collection.
